# Cyclic Peptide-Based Biologics Regulating HGF-MET

**DOI:** 10.3390/ijms21217977

**Published:** 2020-10-27

**Authors:** Hiroki Sato, Ryu Imamura, Hiroaki Suga, Kunio Matsumoto, Katsuya Sakai

**Affiliations:** 1 Division of Tumor Dynamics and Regulation, Cancer Research Institute, Kanazawa University, Kanazawa 920-1192, Japan; hiroki.sato@staff.kanazawa-u.ac.jp (H.S.); imamura@staff.kanazawa-u.ac.jp (R.I.); kmatsu@staff.kanazawa-u.ac.jp (K.M.); 2 WPI-Nano Life Science Institute (WPI-NanoLSI), Kanazawa University, Kanazawa 920-1192, Japan; 3 Department of Chemistry, Graduate School of Science, The University of Tokyo, Tokyo 113-0033, Japan; hsuga@chem.s.u-tokyo.ac.jp; 4 Tumor Microenvironment Research Unit, Institute for Frontier Science Initiative, Kanazawa University, Kanazawa 920-1192, Japan

**Keywords:** atomic force microscopy, cyclic peptide, HGF, MET, MET agonist, PET imaging, synthetic HGF

## Abstract

Using a random non-standard peptide integrated discovery system, we obtained cyclic peptides that bind to hepatocyte growth factor (HGF) or mesenchymal-epithelial transition factor. (MET) HGF-inhibitory peptide-8 (HiP-8) selectively bound to two-chain active HGF, but not to single-chain precursor HGF. HGF showed a dynamic change in its molecular shape in atomic force microscopy, but HiP-8 inhibited dynamic change in the molecular shape into a static status. The inhibition of the molecular dynamics of HGF by HiP-8 was associated with the loss of the ability to bind MET. HiP-8 could selectively detect active HGF in cancer tissues, and active HGF probed by HiP-8 showed co-localization with activated MET. Using HiP-8, cancer tissues with active HGF could be detected by positron emission tomography. HiP-8 seems to be applicable for the diagnosis and treatment of cancers. In contrast, based on the receptor dimerization as an essential process for activation, the cross-linking of the cyclic peptides that bind to the extracellular region of MET successfully generated an artificial ligand to MET. The synthetic MET agonists activated MET and exhibited biological activities which were indistinguishable from the effects of HGF. MET agonists composed of cyclic peptides can be manufactured by chemical synthesis but not recombinant protein expression, and thus are expected to be new biologics that are applicable to therapeutics and regenerative medicine.

## 1. Introduction

Many aspects of the biological and physiological roles of HGF and the MET receptor have been introduced in previous articles [1,2,3]. The notable biological activities driven by the HGF-MET pathway include the induction of dynamic tubular morphogenesis and cell motility into the epithelial cells [4,5]. This dynamic morphogenic activity could not be performed by other growth factors [4], and was attributed to the preferential association of MET with the adapter protein Gab1 [5]. As is the case with other growth factors, HGF promotes the survival of cells, including hepatocytes and neurons. Notably, a target (limb mesenchyme)-derived chemoattractant factor for spinal motor neurons was identified to be HGF [6].

A variety of tissue-specific MET knockout mice have revealed that the HGF-MET pathway promotes tissue regeneration, protects against cell death, and suppresses the progression of chronic inflammation and fibrosis in different tissues and cell types [3]. Therefore, the promotion of MET activation has been reported to facilitate the treatment of injuries and diseases in preclinical models. Clinical trials using the HGF protein for the treatment of patients, including those with fulminant hepatitis, spinal cord injury, amyotrophic lateral sclerosis (ALS), and vocal fold scarring, have been completed or are ongoing. Based on the angiogenic activity of HGF, gene therapy by the intramuscular administration of a plasmid expressing HGF was approved for the treatment of patients with critical limb ischemia in 2019.

**Figure 1:**
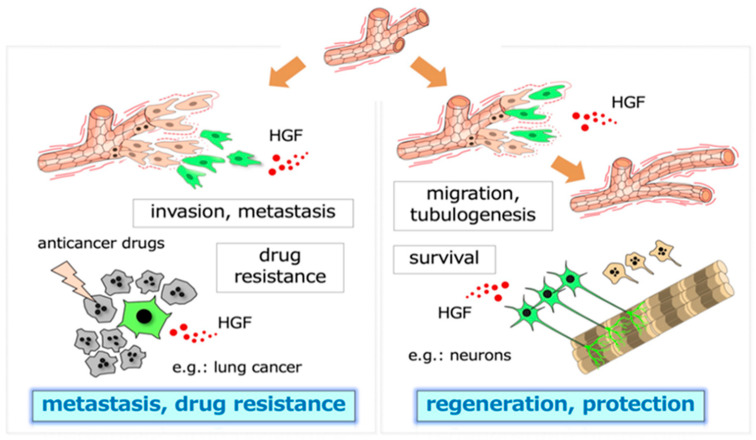
Two-pronged roles of HGF. The dynamic branching morphogenesis (e.g., in renal tubular cells) and the promotion of cell survival (e.g., in neurons, hepatocytes) mediated by the HGF-MET pathway play roles in tissue regeneration and protection after injury (**right** panel). In tumor tissues, similar biological activities, namely, dynamic cell movement and survival, promoted by MET activation participate in invasion–metastasis and therapy resistance against molecular-targeted drugs (**left** panel). The cells responding to HGF are shown in green.

The biological activities that are driven by the HGF-MET pathway all play a role in the acquisition of malignant characteristics by tumor cells, namely, invasion, metastasis, and drug resistance in the tumor microenvironment (Figure 1) [1,2,3]. The selective inhibition of MET activation has been anticipated to become a molecular-targeted therapy of cancer, although no selective inhibitors of HGF-MET have been approved as drugs for cancer. Inhibitors that have advantages over conventional small-molecule tyrosine kinase inhibitors and antibody drugs, and the application of new molecular tools for patient selection are expected for successful clinical trials of the HGF-MET inhibitors.

Macrocyclic peptides (here referred to as ‘cyclic peptides’) have become a hot topic in drug discovery. In the last few years, we have applied macrocyclic peptides to regulate HGF and MET; one is agonistic, and the other is inhibitory. The outstanding specificity and performance of these agonistic or inhibitory macrocyclic peptides for HGF-MET are introduced in this article.

## 2. Cyclic Peptides and RaPID System

The background of the cyclic peptides as drug candidates is as bioactive peptides discovered in nature. A typical example of a macrocyclic peptide drug from nature is cyclosporin A. The cyclosporin A–cyclophilin complex binds to calcineurin, thereby inhibiting the protein phosphatase activity of calcineurin. The constrained structures of cyclic peptides composed of 5–20 amino acids can produce antibody-like binding affinity and specificity [7]. Cyclic peptides have the potential to combine the advantages of both small-molecule drugs and biologics (antibodies, growth factors, enzymes, etc.): they can target a unique chemical space, and exhibit different pharmacokinetic profiles to either small molecules or biologics [7,8,9], thus bridging the gap between small molecules and biologics.

**Figure 2:**
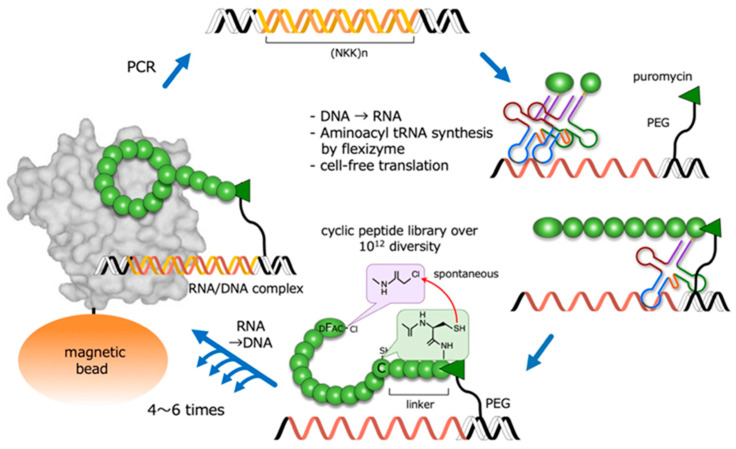
Outline of the RaPID system for the highly efficient screening of macrocyclic peptides from a library with a diversity of over 10^12^ structures. PEG: polyethylene glycol; DFac-Cl: D-chloroacetyl phenylalanine.

In order to synthesize and screen large libraries of cyclic peptides against protein targets, several powerful combinatorial library technologies have been developed. One such technique is random non-standard peptide integrated discovery (RaPID) [10], which integrates mRNA display with flexible in vitro translation (FIT) genetic code reprogramming [11]. It also allows the synthesis of very large (>10^12^) libraries of cyclic peptides that can be readily screened for the ability to bind to protein targets of interest (Figure 2). In the RaPID system, cyclic peptides that bind to target molecules are selected from among cyclic peptides with random sequences. For this, the starting material consists of oligo DNA encoding random peptide sequences (>10^12^). The production of the DNA library is followed by transcription, and the mRNAs are then translated into peptides using ribosome-mediated protein synthesis. By using a ‘flexizyme’—an artificial ribozyme catalyzing the aminoacylation of any tRNA with non-native amino acids—the RaPID system can incorporate non-native amino acids into peptide sequences. Notably, N-terminal non-native modified amino acids, such as D-chloroacetyl phenylalanine, spontaneously react with a cysteine residue at the C-terminal or within a peptide chain, resulting in a covalently-linked cyclic peptide. The repetition of this RaPID cycle is a highly efficient and rapid way to obtain cyclic peptides that bind to target proteins.

## 3. HiP-8 (HGF-Inhibitory Peptide-8)

### 3.1. Background

HGF is biosynthesized and secreted as single-chain HGF (scHGF); the cleavage of scHGF at Arg494–Val495 generates two-chain HGF (tcHGF) [12,13]. scHGF is a biologically inactive precursor which is incapable of activating the MET receptor, while tcHGF is the only active molecular species that can activate MET. In normal tissues, HGF mainly exists as scHGF, while the processing of scHGF into tcHGF occurs in injured tissues [14], indicating that the local processing of scHGF to tcHGF may play an important role in regulating MET receptor activation following a tissue injury. Although the localization of tcHGF and scHGF had not been elucidated, the distinct localization of tcHGF from scHGF was demonstrated recently, using a tcHGF-specific antibody [15]. In the stomach, scHGF is mainly expressed and localized in smooth muscle cells, while tcHGF is mainly localized in the epithelial gland base region. Importantly, tcHGF localization nearly overlaps with the localization of activated MET (pMET), and pMET in the glandular base region is closely associated with the localization of gastric epithelial stem cells, again indicating the importance of tcHGF in MET activation [15].

The proteolytic processing of scHGF to tcHGF is catalyzed by several serine proteases, such as HGF-activator and matriptase [12,16,17]. HGF-activator induces G_0_-to-G_Alert_ cell-cycle transition in stem cells in various tissues in response to tissue injury [18]. Both the HGF-activator-mediated and matriptase-mediated mechanisms are proposed to be involved in the processing of scHGF into tcHGF in cancer tissues [12,19]. Therefore, the selective detection and/or inhibition of tcHGF is particularly important in cancer diagnosis and treatment.

### 3.2. Discovery and Specificity

**Figure 3:**
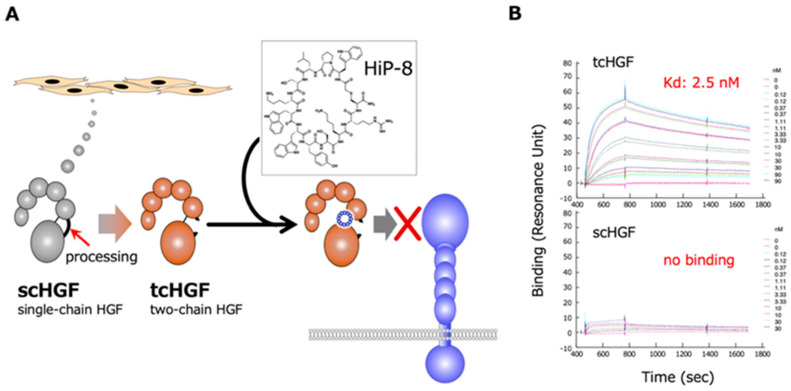
Outline of the action of HiP-8 (**A**) and the selective binding of HiP-8 to two-chain active HGF (**B**). Once HiP-8 binds to tcHGF, tcHGF cannot bind to MET (**A**). The sensorgram shows that HiP-8 selectively binds to tcHGF, but not to scHGF (**B**) [20].

The RaPID system was employed to obtain cyclic peptides that bind to HGF, using human recombinant HGF as bait [20]. Sixteen cyclic peptides that are expected to bind to HGF were chemically synthesized and tested for an inhibitory effect on HGF-induced MET activation/phosphorylation in human cells in culture. Among these cyclic peptides, HiP-8 (HGF-inhibitory Peptide-8) shows the most potent inhibitory activity (Figure 3). HiP-8 is composed of 12 amino acids, and binds tightly to HGF, with a dissociation constant (*K*_D_) of 0.4 nM and a remarkably slow dissociation rate (*k*_off_ = 0.4 × 10^−3^ s^−1^). HiP-8 inhibits HGF-MET interaction with an IC_50_ of 0.9 nM, indicating the excellent ability of HiP-8 to inhibit HGF. Once HiP-8 binds to HGF, HGF bound with HiP-8 cannot bind to the MET receptor. The surface plasmon resonance analysis for the binding of HiP-8 to scHGF and tcHGF clearly shows the selective binding of HiP-8 to tcHGF, but not to scHGF (Figure 3).

### 3.3. Inhibition of Molecular Dynamics

**Figure 4:**
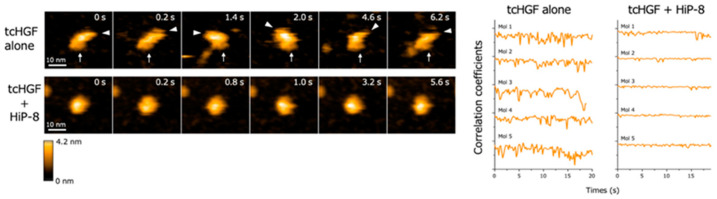
Flexible and dynamic domain movement in tcHGF, and the inhibition of the molecular dynamics of tcHGF by HiP-8 [20]. The left panels show the real-time changes in the molecular shape of tcHGF with or without HiP-8. The lines in the right panels indicate quantitative time-dependent changes in the molecular shape of individual tcHGF molecules. The remarkably changing curves in tcHGF indicate flexible and dynamic changes in molecular shape, while such dynamic changes of tcHGF are inhibited by HiP-8, indicating a change from a dynamic to a static state in tcHGF by HiP-8.

High-speed atomic force microscopy (AFM) enables the real-time observation of macromolecules with nanometer resolution under near-physiological conditions [21,22]. When the real-time changes in structure and dynamics of HGF were analyzed by high-speed AFM, both scHGF and tcHGF showed a rapid movement of domains. Because high-speed AFM can visualize real-time changes in the structure and shape of biological molecules in solution, the result suggests that scHGF and tcHGF are constitutively flexible and dynamic in solution. As is consistent with the lack of binding of HiP-8 to scHGF, no change in the structure and shape of scHGF was seen after the addition of HiP-8 to scHGF. In contrast, HiP-8 inhibited the flexible and dynamic domain movement in the tcHGF molecule (Figure 4). An analysis of the structure–activity relationship indicated that HiP-8 binds to multiple domains of tcHGF, suggesting a multivalent interaction with tcHGF using different faces in the cyclic structure. HiP-8 may bind to a space created by different domains in tcHGF.

### 3.4. Potential Application

**Figure 5:**
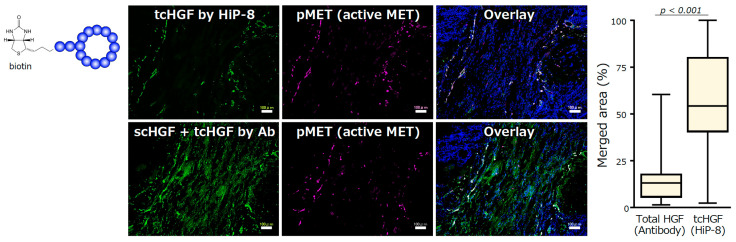
Detection of tcHGF by HiP-8, and the colocalization of HiP-8 probed tcHGF and pMET [20]. Scale bar, 100 μm.

The selective binding to and inhibition of tcHGF by HiP-8 suggests the potential for its use of HiP-8 in cancer diagnosis and/or therapeutics. One of the advantages of the application of the cyclic peptides discovered by the RaPID system is the ability to chemically modify cyclic peptides without interfering with their binding ability, because chemical modifications are carried out at the end of short linker sequences opposite the cyclic peptide part. HiP-8 was conjugated with biotin and used as a molecular tool to detect tcHGF in human cancer tissues (Figure 5). The localization of HGF—as detected by an antibody that recognizes both scHGF and tcHGF—does not overlap with the localization of active MET (pMET). In contrast, the localization of tcHGF—as detected by HiP-8—shows significant overlap with pMET. Therefore, HGF is distributed in cancer tissues mainly as scHGF, and MET activation occurs in cells in the close vicinity of tcHGF generated in the tumor microenvironment. HiP-8 could become a useful molecular tool to detect pMET as well as tcHGF in cancer tissues.

**Figure 6:**
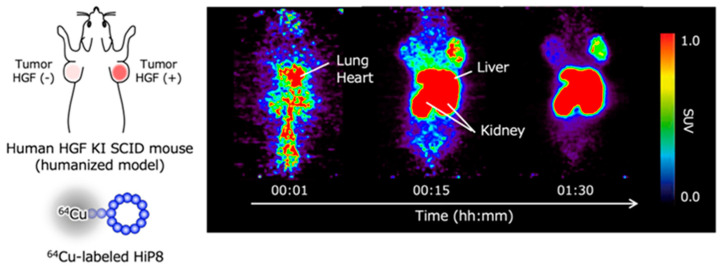
Excellent accumulation of HiP-8 in HGF-high cancer tissues in PET molecular imaging [20].

Positron emission tomography (PET) is widely used for diagnosis by molecular imaging that reflects the characteristics of cells. For instance, PET imaging with 2-[^18^F]fluoro-2-deoxy-D-glucose has been used to detect cancer, based on the characteristic that cancer cells incorporate and utilize glucose more efficiently than normal cells. Because higher levels of HGF, or more precisely higher levels of tcHGF and pMET, are considered to be associated with cancer progressions such as drug resistance, the non-invasive detection of tcHGF and pMET is expected to be important for the selection of therapeutic drugs. When HGF-low and HGF-high lung cancer cells were inoculated in human HGF knock-in mice—in which the endogenous mouse HGF gene was replaced by the human HGF gene and radiolabeled HiP-8 was administered—the efficient accumulation of HiP-8 occurred in HGF-high cancer at a higher level than that in HGF-low cancer (Figure 6) [20]. The clinical trials of drug candidate inhibitors for HGF-MET have been largely disappointing [23,24]. The main reason why previous clinical trials did not achieve their efficacy endpoints is the lack of the appropriate selection of cancer patients in which the cancer growth depends on the activation of MET (pMET). MET gene amplification does not mean MET activation. The HGF levels in cancer tissue are not simply associated with pMET because only tcHGF—but not scHGF, which exists at much higher levels than tcHGF—is a key molecule for the activation of MET. No molecular tools or techniques have been available to non-invasively detect tcHGF and pMET. HiP-8 can be used as an excellent molecular tool to detect tcHGF and perhaps pMET in cancer patients by PET imaging.

## 4. Synthetic MET Agonists Based on Cyclic Peptides

### 4.1. Discovery

**Figure 7:**
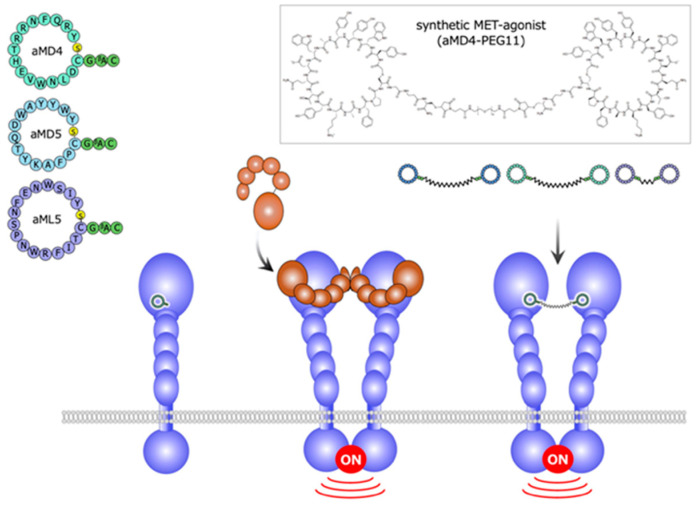
Structures and outline of the activity of a synthetic MET agonist composed of cyclic peptides [25].

**Figure 8:**
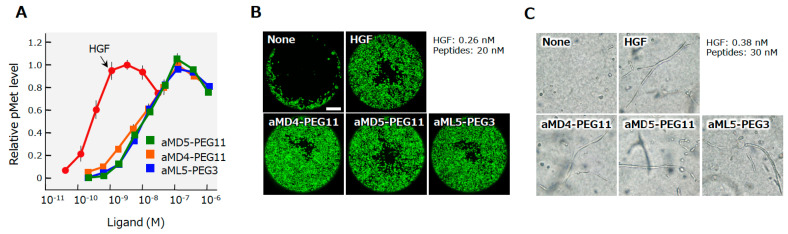
MET receptor activation (**A**), the promotion of cell motility (**B**), and the induction of tubulogenesis (**C**) by HGF and synthetic MET agonists by cyclic peptides [25]. The MET activation, cell motility, and tubulogenesis were analyzed using human mesothelioma cells (**A**) or normal human renal tubular cells (**B**,**C**). Scale bar, 400 μm.

Using the extracellular region of the human MET receptor as bait, the cyclic peptides designated aMD4, aMD5, and aML5 were obtained (Figure 7) [25]. These cyclic peptides bind to the extracellular region of MET (the *K*_D_ values are 2.4, 2.3, and 19.0 nM for aMD4, aMD5, and aML5, respectively), but they do not inhibit HGF-dependent MET activation. Because the dimerization is an essential process for growth factor receptor activation, it is conceivable that, when these cyclic peptides are chemically linked at a certain distance, the cross-linked bivalent MET-binding cyclic peptides activate MET. In actuality, cross-linked MET-binding cyclic peptides (hereafter referred to as ‘synthetic MET agonists’) induce MET dimerization and activate the MET receptor (Figure 7 and Figure 8A) [25].

### 4.2. MET Activation and Biological Activities

The maximal activities for MET activation by these bivalent MET-binding cyclic peptides are comparable to the maximal activity of HGF protein, while the concentrations of the synthetic MET agonists needed to induce maximal MET activation are 20–40 times higher than those of HGF protein [25]. MET activation results in different biological activities depending on the cell type and context. Synthetic MET agonists promote the motility and proliferation of cells, and induce tubulogenesis by epithelial cells when cells are in a 3D collagen matrix (Figure 8B,C). The biological activities of synthetic MET agonists showed activities comparable to those of HGF in different assays.

**Figure 9:**
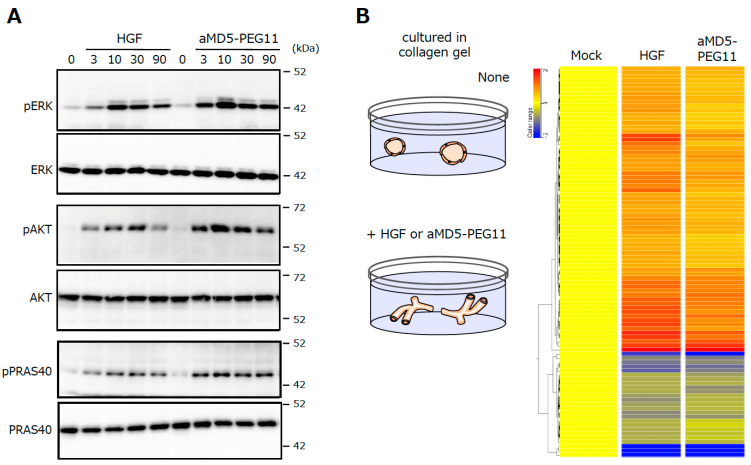
Activation of the intracellular signaling molecules (**A**) and the changes in the gene expression profiles (**B**) induced by HGF and aMD5-PEG11. In **A**, human mesothelioma cells were cultured in 2D conditions. In **B**, human renal tubular cells were cultured in 3D collagen gel. These data were taken from our previous report [26].

The investigation of the activation profiles for intracellular signaling molecules downstream of MET, such as Erk and Akt, and their changes in the gene expression profile upon MET activation indicate that the synthetic MET agonist induces the activation of signaling molecules and changes in gene expression profiles, with a potency that is largely indistinguishable from that of HGF protein (Figure 9) [26]. The performance of synthetic MET agonists composed of cyclic peptides indicates that synthetic agonists for growth factor/cytokine receptors; in other words, synthetic growth factors/cytokines are obtainable basically by the same method.

## 5. HGF-mimetics (MET-agonists) and Potential Applications

### 5.1. MET Agonists with Different Molecular Characteristics

**Figure 10:**
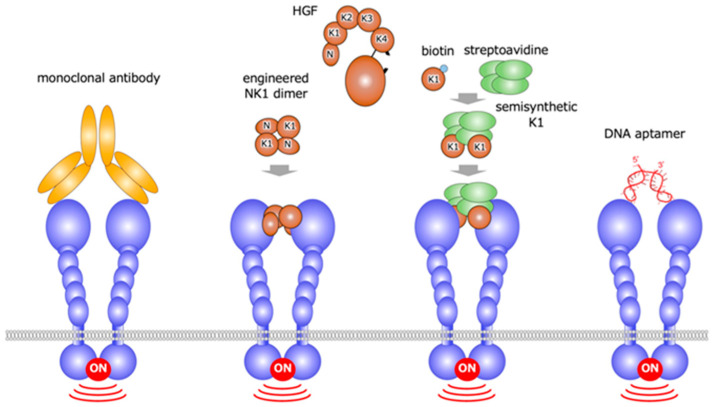
HGF-mimetic (MET-agonistic) molecules created by different approaches: monoclonal antibodies, engineered NK1, semisynthetic K1, and DNA aptamer.

Based on the concept that growth factor receptor activation depends on receptor dimerization, HGF-mimetic molecules, were discovered by different approaches [27,28,29,30,31,32,33,34,35,36]. These approaches include the use of: monoclonal antibodies based on the bivalent characteristic of antibodies [27,28,29,30,31]; the engineered protein eNK1, created by the disulfide-linked NK1 (N-terminal and the first kringle) domains of HGF [32,33]; the semi-synthetic engineered K1 domain, designed to be multimer by biotin-avidin interaction [34]; and DNA aptamer [35,36] (Figure 10).

The MET-agonist monoclonal antibody protects cardiac myocytes from cell death induced by oxidative stress or hypoxia [27,28,29]. In mice, the administration of MET-agonist monoclonal antibody enables us to sustain the survival of transplanted human hepatocytes or human hepatocyte-like cells derived from induced pluripotent stem cells [30,31]. eNK1 was created to archive efficient expression and chemical stability by amino acid replacement in the linker region between the N-terminal and K1 domains, and the eNK1 dimer was prepared through the introduction of N-terminal cysteine residue [32]. In biological assays, the efficacy of eNK1 was comparable to HGF [33]. In the DNA aptamer approach, MET-binding aptamer was obtained, and it was then linked by single strand or double strand linkers [35]. The most potent DNA aptamer is composed solely of unmodified 100-mer single stranded DNA, and it activates MET at EC50 value of 1–5 nM. In a mouse model of fulminant hepatitis, the intravenous administration of a MET-agonistic DNA aptamer increased the MET activation and suppressed the cell death of hepatocytes in the liver [36]. MET agonists created by different approaches are expected to be therapeutic molecules.

### 5.2. Potential Applications

In addition to chemical properties, efficiency, and manufacturing, therapeutic applications of HGF and HGF-mimetic MET-agonists can be considered based on the phenotypes of MET-knock out mice, at least to some extent. Until now, the characteristics of a variety of tissue-specific mice have been noted (Table 1) [37,38]. The representative phenotypes were first seen in *MET* knockout in the liver. Hepatocytes subjected to the selective loss of the functional MET were highly susceptible to cell death even after mild liver injury, indicating that the anti-apoptotic activity of HGF plays a role in the protection of the liver [39]. Liver-specific MET^−/−^ mice showed a delayed liver regeneration that was associated with a persistent inflammatory reaction [40]. The livers in hepatocyte-specific MET^−/−^ mice were more susceptible to chronic inflammation and fibrotic change compared with the control mice [40].

The loss of functional MET in renal tubules caused no appreciable defect in renal function; however, tubular cell-specific Met^−/−^ mice displayed higher serum creatinine and an increase in apoptosis compared with control mice after renal injury [48]. In podocyte-specific MET^−/−^ mice, no pathology was seen, but when they were subjected to a toxic renal injury of the podocytes, these mice developed more severe podocyte apoptosis and albuminurea compared to the control mice [49]. The disruption of the *MET* gene in keratinocytes demonstrated an indispensable role for the HGF-MET pathway in skin wound healing, because the migration of keratinocytes post-wounding was almost completely impaired in MET^−/−^ keratinocytes [52]. HGF-MET signaling is not essential for β-cell growth, but it is essential for normal glucose-dependent insulin secretion and glucose homeostasis [53,54,55]. Mice that were deficient in the MET in their lung alveolar epithelial cells demonstrated impaired airspace formation marked by a reduction in alveolar epithelial cell abundance and survival, the truncation of the pulmonary vascular bed, and enhanced oxidative stress. The HGF-MET pathway plays a definitive role in alveolar formation and protection [64]. MET-deficiency in cardiomyocyte indicates a hypertrophic change in cardiomyocytes and interstitial fibrosis by 6 months, followed by systolic cardiac dysfunction by 9 months [65]. These tissue-selective loss-of-function approaches imply that MET activation may become an important therapeutic for the treatment of diseases in different organs, if MET can be efficiently activated by HGF or MET-agonists for an appropriate duration.

HGF promotes the survival of a variety of cells, including motor neurons. In a model of a spinal cord injury, intrathecally-infused HGF increased the number of viable motor neurons and promoted the recovery of locomotive functions [71]. In a model of ALS, intrathecally-infused HGF increased the number of surviving motor neurons, suppressed disease progression, and promoted the lifespan [72]. Based on these preclinical results, Phase II and Phase III clinical trials of the use of recombinant HGF for the treatment of ALS and spinal cord injury are ongoing, respectively. The half-life of HGF in the blood is short [73,74]; however, in these clinical trials, HGF is administered intrathecally. Intrathecally-administered HGF is expected to be retained in the intraspinal space without rapid leakage into the blood circulation. For the clinical application of recombinant HGF for the treatment of chronic diseases, the combination of HGF with materials for a slow-release delivery seems to be a key. Instead, MET-agonist antibodies have an advantage for the treatment of chronic or cardiovascular diseases because of their long-life stability during blood circulation. If synthetic MET agonists that are stable in the blood circulation could be prepared by chemical modification, they would be expected to be applicable for the treatment of chronic diseases. Using the potentially higher ability of synthetic MET agonists to penetrate tissues because of their small size, synthetic MET agonists may be applicable for local delivery close to injured tissues. In models of brain ischemia, intraventicular HGF protects cerebral neurons, indicating the potential application of MET agonists for the treatment of cerebral diseases. In order to realize this, we need a technical breakthrough to efficiently pass MET-agonists through the blood–brain barrier.

Growth factors have been used as indispensable tools for the growth, differentiation, and expansion of cells derived from stem cells in cell-based regenerative medicine, whereas recombinant growth factors are highly expensive, which has been a major problem in regenerative medicine. Because the approach taken to obtain synthetic MET agonists can be applied to the discovery of synthetic growth factor receptor agonists, growth factors prepared by chemical synthesis are expected to contribute to stem cell preparation in regenerative medicine.

## 6. Conclusions

Using a highly efficient RaPID screening system for cyclic peptide discovery, we discovered (1) HiP-8, an inhibitory macrocyclic peptide which is specific to two-chain active HGF, and (2) synthetic MET agonists/synthetic HGF. These newly discovered molecules have outstanding ability and properties in terms of their specificity, affinity, and action, namely, the inhibition or activation of the HGF-MET system. Thus, a cross-disciplinary approach seems to be expanding biomedical science within the field of growth factors and their receptors, not only in drug discovery, but also in structural biology. Cyclic peptide-based synthetic molecules regulating HGF-MET are expected to contribute to diagnosis, therapeutics, and regenerative medicine.

## Figures and Tables

**Table 1:** Characteristics of Conditional MET Knockout Mice.

Organ	Target Cells	Characteristics	References
Liver	Hepatocytes	Highly susceptible to apoptosis after liver injury. Impairment in recovery from liver necrosis after liver injury. Steatotic change of the liver in aged mice. Decrease in mitotic hepatocytes and delayed regeneration after partial hepatectomy.	[39,40,41,42,43,44,45,46,47]
	Hepatoblastic (oval) cells	Increased apoptosis, decreased migration, and decreased population in hepatoblastic cells. Impaired differentiation into hepatocytes.	
	Kupffer cells α-SMA+/CK19+ cells Bone marrow-derived immune cells	Increased reactive oxygen species and oxidative stress. Earlier and faster progression of steatohepatitis and earlier and stronger progression of fibrosis in dietary model for steatohepatitis.	
Kidney	Tubular cells	Aggravated renal injury and inflammation after acute kidney injury.	[48,49,50,51]
	Podocytes	Severe podocyte injury and apoptosis, and albuminuria after toxic injury.	
	Collecting duct cells	Increased tubular necrosis and interstitial fibrosis following unilateral ureteral obstruction.	
	Ureteric bud	Reduction in nephron number.	
Skin	Keratinocytes	Lack of keratinocyte migration after skin wound. Severe impairment epidermal wound closure.	[52]
Pancreas	β-Cell	Loss of acute-phase insulin secretion in response to glucose, and impaired glucose tolerance. Diminished glucose tolerance and reduced plasma insulin after a glucose challenge. Susceptible to streptozotocin-induced diabetes	[53,54,55]
Nervous system	All neural cells Forebrain neurons Dorsal pallial neurons	Deficit in contextual fear condition. Reduced volume of cortical tissue. Hyperconnectivity in circuit-specific intracortical neurons. Alteration of neuron architecture. Excitatory hyperconnectivity and hypoactivity. Increases proximal and reduces distal apical dendritic branching of neocortical pyramidal neurons in post-pubertal period.	[56,57,58,59,60,61,62,63]
	Cerebral cortex and hippocampus neurons	Enhanced long-term potentiation (LTP) and long-term depression (LTD) at early developmental stages. Reduced LTP and LTD at young adult stage. Larger size in the rostral cortex, caudal hippocampus, dorsal striatum, thalamus, and corpus callosum.	
	Ganglionic eminence	Increased numbers of striatal GABAergic interneurons in the lateral sensorimotor. Delayed procedural learning.	
	Myenteric plexus neurons	Reduced length of neurites and increased bowel injury.	
Lung	Alveolar type II cells	Impaired airspace formation caused by reductions in alveolar epithelial cell growth and survival.	[64]
Heart	Cardiomyocytes	Cardiomyocyte hypertrophy and interstitial fibrosis by 6 months. Systolic cardiac dysfunction by 9 months. Accumulated reactive oxygen species and imbalance in the antioxidant defenses.	[65]
Immune system	Dendritic cells	Failure to emigrate toward lymph nodes during inflammation. Impaired contact hypersensitivity reaction.	[66,67,68]
	Neutrophils	Increased tumor growth and metastasis.	
	T-cells	Acceleration of age-related thymic involution.	
Muscle	Satellite cells	Defective muscle regeneration in response to injury.	[69]
Breast	Mammary epithelial cells	Defects in branching in mammary glands.	[70]

## Abbreviations

AFM	atomic force microscopy
ALS	amyotrophic lateral sclerosis
FIT	flexible in vitro translation
HGF	hepatocyte growth factor
MET	mesenchymal-epithelial transition factor
HiP-8	HGF-inhibitory peptide-8
PET	positron emission tomography
RaPID	random non-standard peptide integrated discovery
scHGF	single-chain HGF
tcHGF	two-chain HGF
